# Comparative Clinical Outcomes of Electrosurgery Versus Other Techniques for Gingival Depigmentation: A Systematic Review

**DOI:** 10.7759/cureus.109842

**Published:** 2026-05-28

**Authors:** Poonam Rai, Shrushti Sukalkar, Mancy Modi, Ashwin Nandakishore, Arvind Shetty, Sanpreet S Sachdev

**Affiliations:** 1 Department of Periodontology, DY Patil University School of Dentistry, Navi Mumbai, IND; 2 Department of Oral Pathology and Microbiology, Bharati Vidyapeeth (Deemed to be University) Dental College and Hospital, Navi Mumbai, IND

**Keywords:** diode laser, electrosurgery, gingival depigmentation, repigmentation, scalpel

## Abstract

Gingival hyperpigmentation is a common esthetic concern, and several depigmentation techniques have been described. Electrosurgery is widely used because of its affordability and good intraoperative hemostasis, but its comparative clinical performance remains unclear. The present systematic review aimed to systematically evaluate the effectiveness of electrosurgery for gingival depigmentation in comparison with other treatment modalities. A systematic review of comparative clinical studies was performed in PubMed, Scopus, ScienceDirect, Embase, Web of Science, Google Scholar, Semantic Scholar, and EBSCOhost from inception to February 2026. Human comparative studies evaluating electrosurgery against other depigmentation techniques and reporting at least one quantitative clinical outcome were included. Data on depigmentation, postoperative pain, healing, complications, and repigmentation were extracted. Risk of bias was assessed using the Cochrane Risk of Bias 2 (RoB-2) tool and the Risk Of Bias In Non-randomized Studies - of Interventions (ROBINS-I) tool. The certainty of evidence was evaluated using the Grading of Recommendations Assessment, Development, and Evaluation (GRADE) approach. Eight comparative studies were included, comprising seven randomized trials and one non-randomized study. Most studies were conducted in India and mainly involved young adults with physiologic gingival pigmentation. Electrosurgery consistently produced satisfactory short-term depigmentation. However, comparisons with diode lasers generally favored lasers for early postoperative pain and healing. Findings versus the scalpel were more balanced, with electrosurgery offering better hemostasis but no clear overall superiority in patient-centered outcomes. Repigmentation was variably reported and showed inconsistent long-term stability. The certainty of evidence ranged from moderate to very low, mainly because of risk of bias, imprecision, and heterogeneity. Electrosurgery is an effective and accessible option for gingival depigmentation, particularly for short-term pigment reduction. However, its postoperative comfort, healing profile, and long-term stability appear less consistent than its immediate clinical effectiveness.

## Introduction and background

Gingival pigmentation is a common clinical finding that presents as brown to black discoloration of the attached gingiva and interdental papillae. In most individuals, it represents a benign physiologic variation, although local and systemic factors may also contribute. Because gingival appearance is an important component of smile esthetics, visible hyperpigmentation can adversely influence facial esthetics, self-perception, and patient confidence [1]. Gingival color is determined by vascularity, epithelial thickness, keratinization, and the presence of pigments, among which melanin is the principal determinant of gingival hyperpigmentation [2]. Melanin is produced by melanocytes in the basal epithelial layer, and the clinical expression of pigmentation usually reflects increased melanocytic activity rather than an increase in melanocyte number [2].

Clinically, gingival pigmentation may be categorized as physiologic or pathologic, and this distinction is important to identify benign esthetic concerns and exclude conditions requiring further evaluation [3]. With increasing awareness of periodontal and smile esthetics, the demand for gingival depigmentation has increased, particularly among patients with a high smile line or excessive gingival display [4]. In these cases, treatment is performed primarily for esthetic and psychosocial benefit.

Several depigmentation techniques have been described, including scalpel scraping, electrosurgery, cryosurgery, gingival abrasion, free gingival grafting, and laser-based approaches [5]. Among these, electrosurgery remains clinically relevant because it is relatively accessible, provides good hemostasis, and offers a clear operative field [6]. However, it is technique-sensitive, and excessive thermal injury may negatively affect postoperative pain and wound healing [6,7]. Repigmentation is another persistent challenge, as recurrence may occur due to the activation of residual melanocytes, incomplete removal of the pigmented basal layer, or migration of melanocytes from adjacent tissues [7,8].

Although comparative clinical studies on electrosurgery are available, the evidence remains difficult to interpret because of heterogeneity in comparator techniques, pigmentation assessment methods, treated sites, reported outcomes, and follow-up periods. In patients with gingival hyperpigmentation, the present systematic review aimed to evaluate whether electrosurgery or electrocautery, compared with other depigmentation modalities, influenced depigmentation effectiveness, postoperative pain, healing, complications, and repigmentation or recurrence.

## Review

Methodology

This systematic review was conducted and reported in accordance with the Preferred Reporting Items for Systematic Reviews and Meta-Analyses (PRISMA) 2020 statement [9]. The review protocol was registered in the International Prospective Register of Systematic Reviews (PROSPERO) under the registration number CRD420261281485.

Review Question and Population-Intervention-Comparator-Outcome-Study Design (PICOS) Framework

The review question was as follows: In patients with gingival hyperpigmentation, how does electrosurgery compare with other depigmentation modalities in terms of pigment reduction, postoperative pain, wound healing, complications, and repigmentation or recurrence?

The review was structured according to the PICOS framework. The population included human participants with clinically evident gingival hyperpigmentation, whether physiologic, racial, or pathologic, involving any gingival site. The intervention was gingival depigmentation performed using electrosurgery or electrocautery, including diathermy-based techniques, loop or needle electrodes, and related electrosurgical ablation methods. Comparators included any other depigmentation modality such as scalpel scraping, surgical stripping, diode laser, cryosurgery, liquid nitrogen, or free gingival grafting. Outcomes of interest were quantitative measures of depigmentation and recurrence, patient-reported pain or discomfort, healing outcomes, complications, and patient satisfaction where numerically reported. Eligible study designs included randomized controlled trials, controlled clinical trials, cohort studies, and case-control studies with a direct comparator arm.

Eligibility Criteria

Studies were included if they had a comparative clinical design, including randomized controlled trials, controlled clinical trials, cohort studies, or case-control studies. They were required to have at least one study arm using electrosurgery, a direct comparison with one or more alternative depigmentation techniques, and a minimum sample size of 10 participants. At least one postoperative assessment performed at or beyond one week was required. Another inclusion criterion was the reporting of at least one quantitative outcome related to depigmentation, recurrence, pain, healing, complications, or satisfaction.

The primary outcome recorded was repigmentation or recurrence, defined as any quantitatively reported return of pigmentation at the longest follow-up available in each study. Secondary outcomes included early improvement in pigmentation, postoperative pain or discomfort at the earliest comparable timepoint, quantitative healing outcomes, and reported complications or adverse events. Studies were excluded if they were reviews, meta-analyses, narrative reviews, editorials, letters, protocols without results, conference abstracts without sufficient extractable data, case reports, case series, non-comparative studies, studies without an electrosurgery arm, studies with fewer than 10 participants, studies without postoperative follow-up of at least one week, or non-human studies. The eligibility criteria were predefined according to the PICOS framework and are presented explicitly in Table 1, with each component specifying the corresponding inclusion and exclusion criteria.

**Table 1:** Eligibility criteria according to the PICOS framework PICOS: Population-Intervention-Comparator-Outcome-Study Design

Component	Inclusion criteria	Exclusion criteria
Population (P)	Human participants with clinically evident gingival hyperpigmentation, including physiologic, racial, or pathologic pigmentation involving any gingival site	Non-human studies; participants without gingival hyperpigmentation
Intervention (I)	Gingival depigmentation performed using electrosurgery/electrocautery, including diathermy-based techniques, loop or needle electrodes, and related electrosurgical ablation methods	Studies not using electrosurgery/electrocautery as an intervention arm
Comparator (C)	Any other depigmentation modality, such as scalpel scraping, surgical stripping, diode laser, cryosurgery, liquid nitrogen, or free gingival grafting	Studies without a direct comparator group
Outcomes (O)	Quantitative outcomes related to depigmentation, recurrence/repigmentation, postoperative pain/discomfort, healing, complications, or patient satisfaction; at least one postoperative assessment at or beyond one week	Studies without quantitative clinical outcomes of interest; studies without postoperative follow-up of at least one week
Study design (S)	Randomized controlled trials, controlled clinical trials, cohort studies, and case-control studies; minimum sample size of 10 participants	Reviews, systematic reviews, meta-analyses, narrative reviews, editorials, letters, protocols without results, conference abstracts without sufficient extractable data, case reports, case series, non-comparative studies, and studies with fewer than 10 participants

Search Strategy

A comprehensive electronic search was performed in PubMed, Scopus, ScienceDirect, Embase, and Web of Science from database inception to February 2026. Grey literature and supplementary sources were searched through Google Scholar, Semantic Scholar, and EBSCOhost, with screening restricted to the first 300 results sorted by relevance where applicable. The search strategy combined controlled vocabulary, when available, with free-text keywords and Boolean operators related to the main review concepts: gingival pigmentation or depigmentation; electrosurgery, electrocautery, or diathermy; and comparator techniques such as scalpel, laser, cryosurgery, liquid nitrogen, and free gingival grafting. Search terms were combined using operators such as AND and OR, and study design-related terms were applied where appropriate to improve the retrieval of comparative clinical studies. Searches were finalized in February 2026, and records from all sources were exported and merged for centralized de-duplication. The complete electronic search strategies for the major databases, including full search strings, keywords, Boolean combinations, and any filters applied, are provided in the Appendices.

To improve completeness, manual citation searching was performed by screening the reference lists of all included studies, and forward citation tracking was undertaken using Scopus and Web of Science. In addition, Elicit (Ought, Oakland, California, United States) was used as a supplementary identification tool for potentially relevant records. Final eligibility decisions, however, were based only on the predefined inclusion and exclusion criteria after full-text assessment.

Study Selection

All retrieved records were imported into the Elicit systematic review platform. Duplicate records were removed using a two-step approach consisting of automated duplicate identification followed by manual verification using title, authorship, publication year, Digital Object Identifier, journal details, and, when required, full-text comparison. Study selection was completed in two stages: title and abstract screening, followed by full-text assessment of potentially eligible records. Screening was performed by two reviewers (SS and AN) working independently against the predefined eligibility criteria. Any disagreements regarding study eligibility were resolved through discussion and consensus or by consultation with a third reviewer (PR). Reasons for exclusion at the full-text stage were recorded under predefined categories, including wrong intervention, wrong study design, inadequate sample size, insufficient follow-up, absence of quantitative outcomes, or lack of a comparator group.

Data Extraction

A structured data extraction form was prepared before data collection and pilot-tested on two included studies to ensure consistency and completeness. The finalized form was then applied to all eligible studies, and the data extraction was performed using the Elicit systematic review tool, which was then manually verified by the reviewers, AN and PR, who corrected any discrepancies or missing data. Extracted variables included study identification details, country and setting, study design, unit of allocation, sample size, participant characteristics, gingival sites treated, baseline pigmentation characteristics, intervention details, comparator details, adjunctive postoperative care, reported outcomes, follow-up schedule, and the longest available follow-up point. Outcome data extracted included pigmentation scores or indices, recurrence or repigmentation data, pain or discomfort scores, healing outcomes such as epithelialization time or healing indices, complications, and patient satisfaction when numerically reported. All data were recorded exactly as presented in the original studies, including the original scales, units, and timepoints. When data were missing, incompletely reported, or presented only narratively in the original studies, no statistical imputation was attempted, and the findings were synthesized descriptively on the basis of the available published information.

Risk of Bias Assessment

Risk of bias in randomized trials, including split-mouth randomized studies, was assessed using the Cochrane Risk of Bias 2 (RoB-2) tool [10]. The following domains were evaluated: bias arising from the randomization process, bias due to deviations from intended interventions, bias due to missing outcome data, bias in the measurement of outcomes, and bias in the selection of the reported result. Each domain was judged as low risk, some concerns, or high risk, and an overall judgment was generated based on the ratings of the individual domains. For non-randomized comparative studies, risk of bias was assessed using the Risk Of Bias In Non-randomized Studies - of Interventions (ROBINS-I) tool [11]. The evaluated domains included confounding, participant selection, intervention classification, deviations from intended interventions, missing data, outcome measurement, and selection of the reported result. Each study was graded as having low, moderate, serious, or critical risk of bias.

Data Synthesis

Because substantial heterogeneity was anticipated across the included studies in terms of comparator techniques, outcome measures, measurement scales, and follow-up schedules, a narrative synthesis was planned as the primary method of evidence synthesis. Studies were grouped descriptively according to comparator type and major clinical outcomes, including depigmentation effectiveness, postoperative pain, healing, complications, and repigmentation or recurrence. Meta-analysis was considered only if at least two studies were sufficiently comparable in terms of intervention, comparator, outcome, and timepoint. A formal narrative assessment of heterogeneity was undertaken across study design, comparator intervention, pigmentation indices, outcome definitions, and follow-up schedules. A random-effects meta-analysis was performed for outcomes commonly reported across studies at common timepoints. Because all studies assessed the same construct but reported pain scores with minor variation in scaling and reporting format, standardized mean differences with 95% confidence intervals were used. Statistical heterogeneity was assessed using the I² statistic and Cochran's Q test. All other outcomes and comparisons that were unsuitable for pooling because of heterogeneity in study design, comparator techniques, outcome definitions, and follow-up schedules were discussed as subgroups qualitatively.

Certainty of Evidence

The certainty of evidence for major outcomes was assessed using the Grading of Recommendations Assessment, Development, and Evaluation (GRADE) approach [12]. Certainty was assessed for recurrence or repigmentation, improvement in pigmentation, pain, and healing. Randomized evidence initially started at high certainty, whereas non-randomized comparative evidence started at low certainty. The certainty rating was downgraded where appropriate based on risk of bias, inconsistency, indirectness, imprecision, and potential publication bias. A summary of findings table was planned for the principal outcomes. Because no meta-analysis was performed, certainty assessments were based on the body of evidence available for each comparison and outcome in narrative form rather than on pooled quantitative estimates.

Results

**Figure 1:**
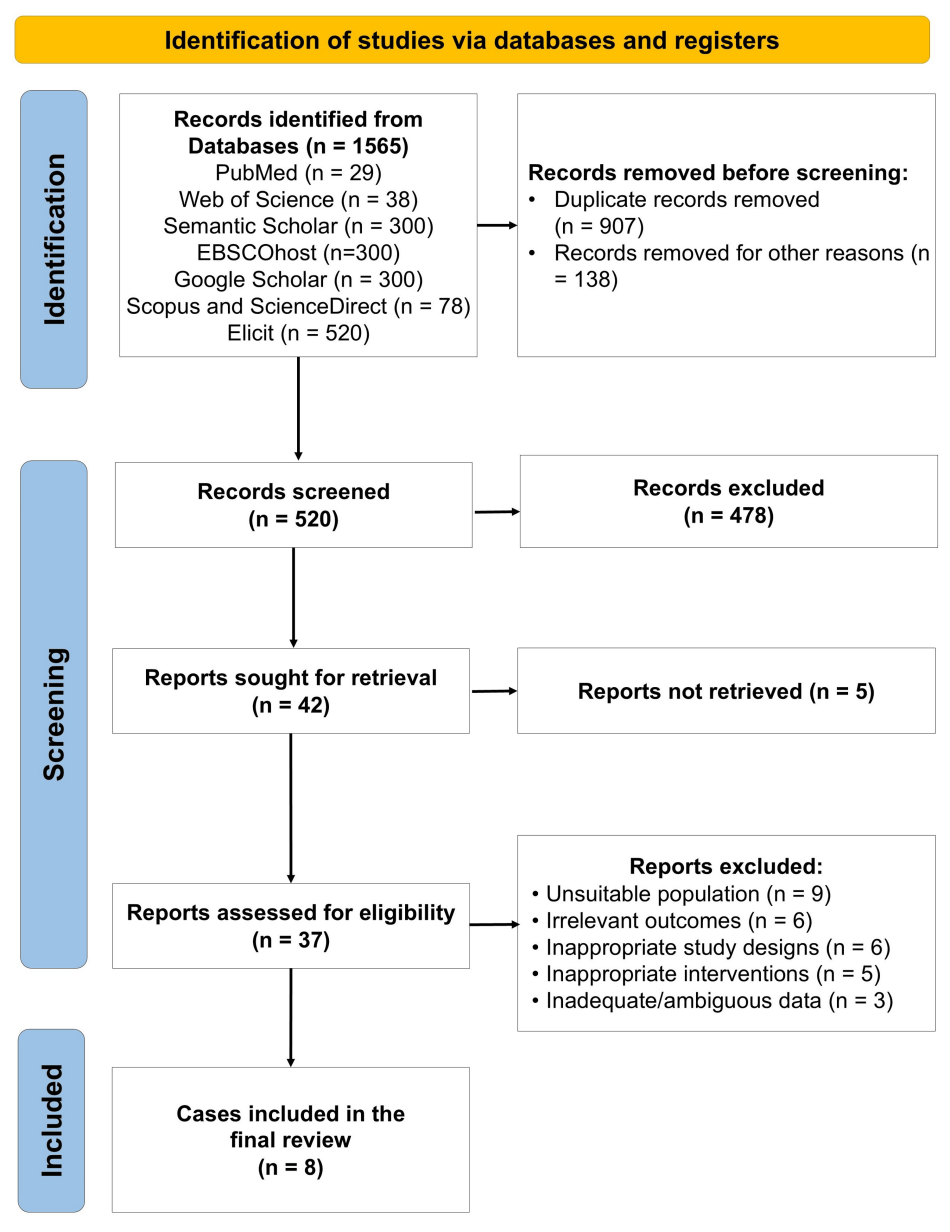
PRISMA flow diagram depicting the study selection process PRISMA: Preferred Reporting Items for Systematic Reviews and Meta-Analyses

The literature search and study selection process identified eight comparative clinical studies that met the predefined eligibility criteria for inclusion in the review [13-20]. These studies formed the final evidence base for qualitative synthesis, with limited quantitative pooling feasible only for postoperative pain in the electrosurgery versus diode laser subgroup. For the broader review questions, quantitative pooling was not appropriate because of marked heterogeneity in design, comparators, outcome measures, and follow-up schedules. The screening flow is presented in Figure 1. The data extracted from these studies is summarized in Table 2.

**Table 2:** Data related to the study characteristics and outcomes of the included records in the present systematic review RCT: randomized controlled trial; OPI: oral pigmentation index; HMI: Hedin melanin index; VAS: Visual Analog Scale; LA: local anesthesia; CHX: chlorhexidine; MGJ: mucogingival junction; FGG: free gingival graft; LNAGD: liquid nitrogen-assisted gingival depigmentation; ECAGD: electrocautery-assisted gingival depigmentation; NR: not reported; BP: Bard-Parker; NS: not significant; HF: high frequency; N₂O: nitrous oxide; CI: confidence interval

Study (year)	Country	Design	Sample size; groups	Age; sex	Baseline pigmentation (index/score; site; type)	Intervention arms (key operative details)	Pigmentation outcome/comparative effectiveness	Pain/discomfort	Healing/complications	Repigmentation/recurrence	Follow-up/timepoints
Chandna and Kedige (2015) [13]	India	RCT	n=20; 2 arms (10/10)	20-40; M12 and F8	Grade 2; anterior maxillary + mandibular; physiological	Electrosurgery: needle + loop electrodes; light brushing strokes; topical lignocaine; device/settings NR. Diode laser: 980 nm, 2-3 W gated-pulsed; fiberoptic handpiece; brush-stroke; topical lignocaine	Treated areas reported "similar in color" to the surrounding gingiva; comparative pigmentation indices NR	VAS: 24-hour laser < electrosurgery (p<0.001); 1 week NS; no analgesics prescribed	Keratinization completed by about 4 weeks; 2 electrosurgery patients had pain on eating/drinking	Long-term repigmentation NR	Intra-op; 24 hours; 1 week (clinical keratinization described at 4 weeks)
Gupta et al. (2014) [15]	India	Split-mouth RCT	n=15; 2 arms (within-patient)	17-25; M6 and F9	Dummett-Gupta OPI: dark 2.81±0.15; wheatish 2.14±0.40; fair 1.54±0.18; visible on smile/speech; physiological	Scalpel scraping: #15 BP; infiltration LA; epithelium at the papilla tip to MGJ. Electrosurgery: loop electrode; light brushing strokes; settings NR; post-op analgesics + antibiotics + 0.12% CHX	Complete depigmentation in both; electrosurgery fewer recurrence sites (4 vs. 7) at 15 months	VAS (2 hours, 24 hours): electrosurgery higher pain at 24 hours (p<0.05)	Scalpel faster healing; electrosurgery delayed/incomplete epithelialization	Recurrence by 15 months: scalpel 7 sites; electrosurgery 4 sites; "patchy"	Days 7/8; 12/13; 21/22; 30/31; 3, 6, 12, and 15 months
Gufran (2016) [16]	Saudi Arabia	RCT	n=18; 2 arms (sizes NR)	18-30; M16 and F2	Dummett-Gupta OPI 3.0 both arms; anterior sextant; physiological	Scalpel: #15 BP; LA; epithelium + underlying pigmented layer. Electrocautery: loop electrode; cautious ablation; dressings removed at 1 week; analgesics given	Depigmentation scores (tables) showed no significant difference between techniques at 1 and 6 months; esthetics "satisfactory"	Pain scale/values NR (analgesics used)	No complications reported (no pain/infection/bleeding/scarring)	No repigmentation at 1 and 6 months	Baseline; 1 month; 6 months (monthly monitoring beyond 6 months mentioned)
Suryavanshi et al. (2017) [17]	India	Non-randomized trial	n=40; 4 arms (10 each)	Age/sex NR	Dummett-Gupta OPI + pigmented area (mm²); anterior; physiological/racial	Scalpel: split-thickness flap; LA. Electrosurgery: HF current + loop electrode; interval technique; LA. Comparators: FGG, diode laser; pre-rinse 0.2% CHX	Residual/repigmentation (%) at 3 months: scalpel 18.57; electrosurgery 19.66; FGG 0; laser 1.6; patient satisfaction "improved color"	VAS: day 2 pain moderate (scalpel/electrosurgery), severe day 2 with FGG; slight/none with laser	Laser healing slightly delayed; FGG healing most delayed; scalpel bleeding described as unpleasant	Quantified at 3 months (percent area)	Baseline; 24 hours; 1 week; 6 weeks; 3 months
Asker et al. (2020) [18]	Egypt	Split-mouth RCT	n=10; 2 arms (within-patient)	18-30 (mean 21.5±3.5); M4 and F6	Dummett-Gupta OPI median 2.0 (1-3); anterior maxillary sites; physiological	Electrosurgery: Surtron diathermy 50D + LA; tissue removal with sterile gauze. Cryosurgery: N₂O probe -75°C, no anesthesia; debridement with tongue depressor; CHX care	Both effective; mild residual pigmentation in 2 cases each; no clear intergroup difference	Pain scores NR; cryosurgery described as painless; electrosurgery required LA	Normal healing; minimal scarring/bleeding	No significant recurrence at 3 and 6 months; mild residual pigmentation in 2 cases each (not esthetically significant)	Baseline; 3 months; 6 months
Jagannathan et al. (2020) [14]	India	RCT	n=30; 3 arms (10/10/10)	24-38; M15 and F15	Dummett-Gupta OPI 3 (uniform); maxillary canine-canine; physiological	Scalpel: #15 BP; LA with adrenaline; epithelium between the papilla tip and MGJ. Electrosurgery: loop electrode + topical gel; settings NR. Diode laser: 940 nm, 1.0 W; pulse interval 0.20 ms; pulse length 0.05 ms; topical gel; adjuncts paracetamol + CHX	Laser: faster healing + least pain; procedure time: scalpel 28:30 vs. electrosurgery 17:20; direct electrosurgery vs. scalpel significance NR	VAS: laser lowest across timepoints; significant differences among groups; scalpel/electrosurgery higher at 2 hours and 24 hours, improving by 1 week	Laser had the least healing time; scalpel/electrosurgery delayed epithelialization; complications NR	Repigmentation at 14 months: scalpel 80%, electrosurgery 70%, laser 20%	Baseline; 24/48 hours; weeks 1-4; 3, 6, 12, and 14 months
Kamboj and Salaria (2020) [19]	India	Split-mouth RCT	n=16; 2 arms (within-patient)	15-30; M13 and F3	Dummett-Gupta OPI grade 3; HMI grade 4; labial/buccal physiological GMP (#14-24; #34-44)	LNAGD: liquid nitrogen 20 seconds after 15% lidocaine. ECAGD: Bonart unit; mode 1; 3RF/2 MHz; fine wire + loop electrode; CHX; analgesics (none required)	Both significantly reduced pigmentation (p<0.05); healing score LNAGD 13 ("excellent") vs. ECAGD 22 ("good"); LNAGD superior reduction in melanocyte density	VAS: pain significant up to 1 day; significant between-group difference at 0-1-day interval; no analgesics required	LNAGD: de-epithelialization by 96 hours; re-epithelialization completed by about 2 weeks. ECAGD: re-epithelialization about 3-4 weeks	Repigmentation at 3 months: 4/16 sites (about 25%)	Baseline; 0 hours; 1 day; 7 days; 8/24/72/96 hours; weeks 1-4; 3 months
Yadav et al. (2022) [20]	India	Parallel RCT	n=40; 2 arms (20/20)	Age/sex NR	Gupta et al. criteria; moderate-severe; bimaxillary anterior; pathological hyperpigmentation	Electrosurgery: technique described; device/settings NR. Diode laser: details NR; setting NR	Both effective; healing at 1 month uneventful in all laser cases vs. 15 electrosurgery cases; healing scores favored laser; repigmentation NS	VAS: electrosurgery 2.70±1.34 (intra-op), 3.10±1.62 (24 hours), 0.70±0.77 (7 days); laser 0.70±0.73, 1.60±0.82, 0.35±0.49; between-group pain NS at 7 days	Healing assessed at 1 and 3 months; laser slight edge; complications NR	Repigmentation: electrosurgery 4 cases; laser 3 cases (NS)	Intra-op; 24 hours; 7 days; 1 month; 3 months

Study Characteristics

The included studies comprised eight comparative investigations evaluating electrosurgery for gingival depigmentation, of which seven were randomized trials and one was a non-randomized study. Sample sizes ranged from 10 to 40 participants, and most studies enrolled young adults with physiological pigmentation involving the anterior gingiva. Six studies were conducted in India, while one each originated from Egypt and Saudi Arabia [13-20]. Electrosurgery was compared with scalpel scraping, diode laser, cryosurgery, liquid nitrogen, or free gingival grafting. Baseline pigmentation was most commonly assessed using the Dummett-Gupta oral pigmentation index (DOPI), but other approaches included simple clinical grade-based scoring, the oral pigmentation index (OPI), the Hedin melanin index (HMI), investigator-defined criteria based on the classification by Gupta et al., and combined methods that paired an index score with measurement of pigmented area in square millimeters. These measures were not fully interchangeable because some assessed ordinal clinical severity, whereas others also captured the extent of pigmentation, thereby contributing to methodological heterogeneity across studies. Reporting of operative parameters was frequently incomplete. Although some studies described selected technical details such as laser wavelength, power settings, or electrode type, many did not report electrosurgical settings, current characteristics, application intervals, pulse parameters, or protocol standardization in sufficient detail, thereby limiting reproducibility and making it more difficult to interpret inter-study differences in pain, healing, and recurrence outcomes.

A formal narrative assessment of heterogeneity is presented in Table 3. Considerable clinical and methodological heterogeneity was observed across study design, comparator technique, baseline pigmentation measures, outcome definitions, and follow-up schedules, which precluded meta-analysis and limited the synthesis to a structured narrative approach.

**Table 3:** Formal narrative assessment of heterogeneity across the included studies RCT: randomized controlled trial; DOPI: Dummett-Gupta oral pigmentation index; OPI: oral pigmentation index; HMI: Hedin melanin index

Domain of heterogeneity	Variability observed across the included studies	Examples from the included studies	Why pooling was not appropriate
Study design and unit of allocation	Parallel-arm randomized controlled trials, split-mouth randomized controlled trials, and one non-randomized four-arm comparative study were included	Parallel designs [13,15,18,20]; split-mouth designs [14,17,19]; non-randomized four-arm study [16]	These designs generate different effect structures, and split-mouth correlation data were not reported consistently
Comparator intervention	Electrosurgery was compared with diode laser, scalpel scraping, cryosurgery, liquid nitrogen-assisted gingival depigmentation, and free gingival grafting	[13,14,16-20]	The absence of a uniform comparator prevented clinically homogeneous pooling
Baseline pigmentation assessment	Baseline pigmentation was assessed using grade-based scoring, DOPI, OPI, HMI, Gupta et al.-based criteria, and pigmented area in square millimeters	[13-20]	Baseline severity and treatment response were measured on non-equivalent scales
Outcome definition and reporting	Depigmentation was reported qualitatively, by index change, or by residual pigmented area; recurrence was reported as recurrent sites, recurrent cases, or percentage repigmentation; healing was often described narratively	[13-20]	Common effect estimates could not be derived consistently across studies
Follow-up schedule	Follow-up timepoints ranged from intraoperative or 24–48 hours to 1 week, 1-6 months, and 12-15 months	[13-20]	There was no common follow-up window across all studies for valid pooled comparison
Operative parameter reporting	Electrosurgical settings, electrode characteristics, laser wavelength/power or pulse details, cooling intervals, and procedural standardization were variably and often incompletely reported	[13-20]	Limited reproducibility and uncertainty regarding technique delivery made it difficult to determine whether observed outcome differences reflected true modality effects or differences in operative execution

Depigmentation Effectiveness

Across all included studies, electrosurgery produced clinically appreciable depigmentation and was consistently effective in reducing visible gingival pigmentation during short-term follow-up, which ranged from the immediate postoperative assessment to six months and was most commonly reported up to 1-3 months. However, the degree of quantitative reporting varied considerably, which limited direct comparison across studies. In general, electrosurgery showed effectiveness comparable to scalpel techniques in early follow-up periods, while comparisons with diode laser suggested similar pigment reduction but with some advantage for the laser in overall clinical recovery. In the limited comparisons with cryosurgical and liquid nitrogen-based approaches, electrosurgery also demonstrated meaningful pigment reduction, although these alternative modalities occasionally showed more favorable short-term outcomes.

Pain, Healing, and Complications

Postoperative pain was assessed in most studies, commonly using the Visual Analog Scale. A recurring trend across the evidence base was that electrosurgery tended to be associated with greater early postoperative pain than diode laser, particularly within the first 24 hours. Comparisons with a scalpel were less consistent, although electrosurgery generally did not show a clear advantage in comfort. Healing outcomes were reported in all studies and suggested that electrosurgery may be associated with slightly delayed epithelialization when compared with scalpel, diode laser, or liquid nitrogen in some trials. Despite this, major complications such as infection, uncontrolled bleeding, or scarring were uncommon, and adverse events were largely limited to transient discomfort and delayed healing in selected cases.

Repigmentation and Recurrence

Repigmentation was one of the most clinically important but most variably reported outcomes. Six studies provided recurrence-related data in different formats, including percentage repigmentation, the number of recurrent sites, or the number of affected cases. The findings indicated substantial heterogeneity. Most studies evaluated recurrence only within short-term follow-up periods of 3-6 months [16-20], whereas only two studies extended follow-up to 12 months or beyond [14,15]. Some studies reported no repigmentation during the short-term follow-up, whereas others demonstrated considerable recurrence at longer follow-up intervals of 12-15 months. Overall, electrosurgery appeared capable of providing acceptable short-term esthetic improvement, but the durability of results remained uncertain because recurrence appeared to be time-dependent and longer-term evidence was limited.

Electrosurgery Versus Diode Laser

The largest and most interpretable subgroup consisted of studies comparing electrosurgery with diode laser [13,14,17,20]. Within this subgroup, both modalities generally achieved satisfactory short-term depigmentation, but diode laser showed a more consistent advantage in early postoperative comfort and healing. Lower pain scores at 24 hours and more favorable early healing patterns were reported more consistently in the laser groups, whereas electrosurgery was associated with greater early discomfort and, in some studies, relatively delayed epithelialization [13,14,20]. Recurrence findings within this subgroup were less consistent because follow-up periods differed substantially, ranging from three months to 14 months. Overall, this subgroup provided the clearest pattern in the review, suggesting that although electrosurgery is effective for pigment removal, diode laser may offer a more favorable early postoperative profile.

Electrosurgery Versus Scalpel

The subgroup comparing electrosurgery with scalpel included three studies [15-17]. In this subgroup, both techniques were generally effective in reducing gingival pigmentation in the short term. However, the comparative pattern was more balanced than that observed for diode laser. Electrosurgery offered procedural advantages related to hemostasis and a cleaner operative field, whereas scalpel-treated sites tended to show faster superficial healing and, in some studies, lower early postoperative discomfort [15,16]. Recurrence findings were variable: one study suggested fewer recurrent sites after electrosurgery at longer follow-up, whereas others either reported no recurrence within shorter observation periods or did not demonstrate a clear long-term advantage [15-17]. Thus, within the scalpel subgroup, the evidence did not support a uniformly superior technique and suggested a trade-off between intraoperative control and postoperative recovery.

Electrosurgery Versus Cryosurgery or Liquid Nitrogen-Based Depigmentation

The subgroup comparing electrosurgery with cryosurgery or liquid nitrogen-assisted depigmentation was represented by two studies [18,19]. Both approaches were effective in reducing pigmentation, but cryogenic techniques appeared to offer some advantages in terms of early healing, reduced pain, or both. At the same time, the evidence within this subgroup remained limited because the studies used different designs, reported outcomes differently, and evaluated recurrence over relatively short follow-up periods [18,19]. Accordingly, although cryogenic approaches appeared promising in comparison with electrosurgery, the evidence was too limited and methodologically diverse to support a strong comparative conclusion.

Electrosurgery Versus Free Gingival Graft

Only one non-randomized study included a free gingival graft comparator [17]. In that study, free gingival grafting showed no repigmentation at three months, whereas localized recurrence was observed after electrosurgery. However, the graft procedure was also associated with greater postoperative morbidity and a more technique-sensitive surgical course [17]. Because this comparison was informed by a single non-randomized study with a short follow-up, it should be interpreted cautiously and cannot support broad conclusions regarding comparative superiority.

Overall Interpretation of Subgroup-Based Synthesis

Taken together, the subgroup-based synthesis shows that the evidence was not only heterogeneous in methodology but also clinically fragmented across different treatment comparisons. The most consistent comparative pattern was observed in the electrosurgery versus diode laser subgroup, where diode laser was more often favored for early postoperative pain and healing outcomes. By contrast, the electrosurgery versus scalpel subgroup showed a more balanced trade-off, while comparisons with cryogenic techniques and free gingival grafts were supported by fewer and more methodologically diverse studies. These subgroup-specific differences reduce overall comparability across the full evidence base and weaken the strength of generalized conclusions.

Meta-Analysis

**Figure 2:**
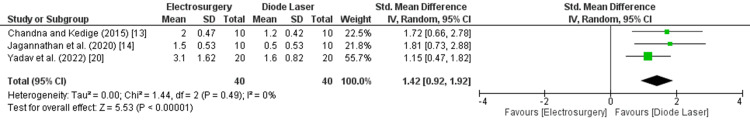
Forest plot showing the limited random-effects meta-analysis of randomized controlled trials comparing electrosurgery versus diode laser for postoperative pain at 24 hours

**Figure 3:**
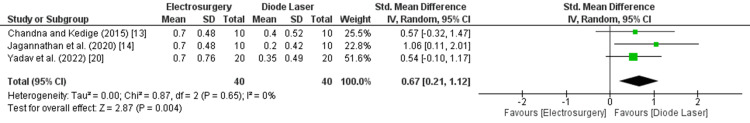
Forest plot showing the limited random-effects meta-analysis of randomized controlled trials comparing electrosurgery versus diode laser for postoperative pain at one week

A random-effects meta-analysis was performed for postoperative pain in the electrosurgery versus diode laser subgroup at 24 hours and one week. Three randomized controlled trials contributed to this analysis. At 24 hours (Figure 2), pooled analysis showed significantly greater pain in the electrosurgery group than in the diode laser group, favoring diode laser, with a large effect size and no observed statistical heterogeneity (standardized mean difference: 1.42; 95% CI: 0.92 to 1.92; I²=0%). At one week (Figure 3), pooled analysis continued to favor diode laser, although the magnitude of difference was smaller than at 24 hours, and again no statistical heterogeneity was observed (standardized mean difference: 0.67; 95% CI: 0.21 to 1.12; I²=0%). These pooled findings support the narrative pattern observed across individual studies that diode laser is associated with more favorable early postoperative comfort than electrosurgery.

Risk of Bias

**Figure 4:**
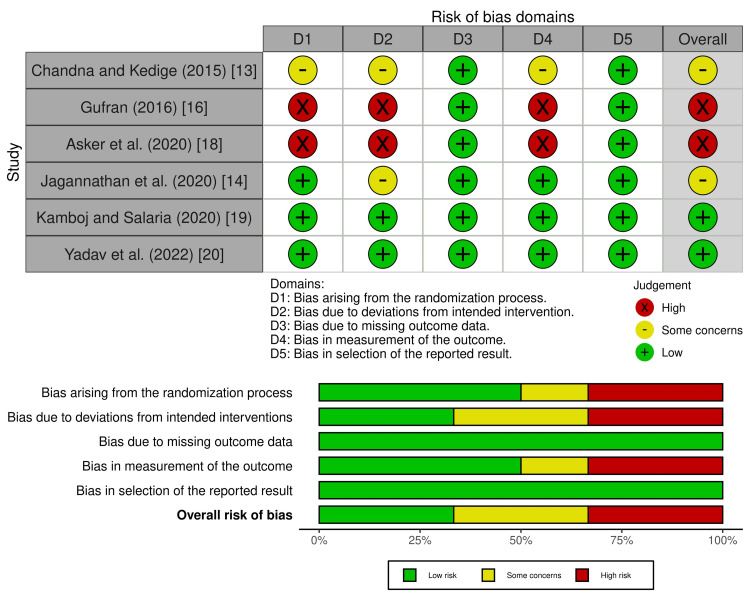
RoB-2 domain-level judgments for the included randomized controlled trials RoB-2: Cochrane Risk of Bias 2

**Figure 5:**
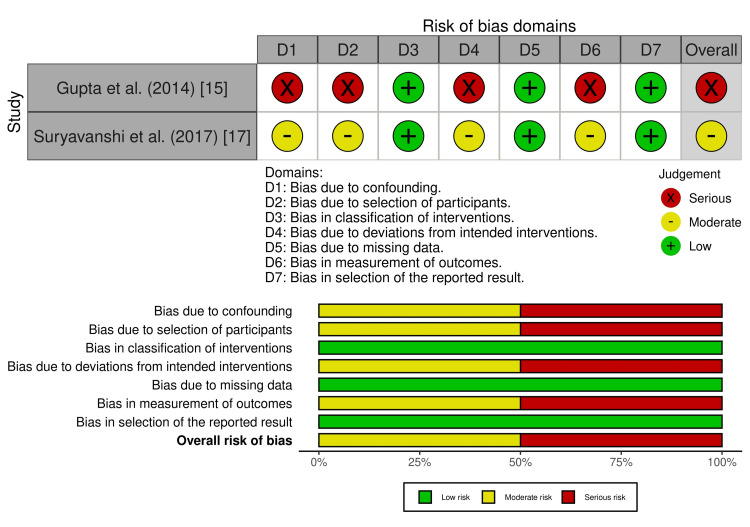
ROBINS-I domain-level judgments for the included non-randomized clinical studies ROBINS-I: Risk Of Bias In Non-randomized Studies - of Interventions

Among the randomized studies, risk of bias ranged from low to high, with the most common concerns involving incomplete reporting of randomization procedures, lack of blinding, and potential subjectivity in outcome measurement. Domain-level assessment for randomized trials is shown in Figure 4. For the non-randomized comparative evidence, the overall risk of bias ranged from moderate to serious, primarily due to confounding and participant selection issues; these assessments are presented in Figure 5.

Certainty of Evidence

Using the GRADE approach, the certainty of evidence ranged from moderate to very low depending on the comparator and outcome considered. Evidence comparing electrosurgery with a diode laser was judged to be low for postoperative pain and healing outcomes, while certainty for recurrence outcomes was very low due to inconsistency, imprecision, and heterogeneity in follow-up periods (Table 4). For electrosurgery versus scalpel, certainty was also low to very low, reflecting small study sizes and limited quantitative reporting. The summary of certainty ratings and main reasons for downgrading is presented in Table 5.

**Table 4:** GRADE summary of findings for electrosurgery vs. diode laser GRADE: Grading of Recommendations Assessment, Development, and Evaluation; RoB: risk of bias; VAS: Visual Analog Scale

Outcome	Studies (participants)	Direction of effect (narrative)	Certainty of evidence (GRADE)	Main reasons for downgrading
Postoperative pain (≤24 hours to 7 days; VAS)	3 (80)	Laser generally showed lower early pain, most consistently at about 24 hours; by 7 days, pain tended to decrease in both arms [13,14,20]	Low	Serious RoB (lack of blinding in several trials); serious imprecision (small total n; no pooled effect/CIs)
Healing (early wound healing/keratinization; about 1-4 weeks)	3 (80)	Laser tended to show more favorable/uneventful healing or faster healing trajectories in comparative descriptions with limited quantitative healing metrics across all trials [14,20]	Low	Serious RoB; serious imprecision (outcome reporting often qualitative; small n)
Repigmentation/recurrence (≥1-14 months)	2 (60)	Findings were inconsistent across follow-up windows: substantial recurrence differences at longer follow-up in one study, but small/non-significant differences at short follow-up in another [14,20]	Very low	Serious RoB; serious inconsistency (different follow-up horizons with conflicting signals); serious imprecision

**Table 5:** GRADE summary of findings for electrosurgery vs. scalpel surgical scraping GRADE: Grading of Recommendations Assessment, Development, and Evaluation; RoB: risk of bias

Outcome	Studies (participants)	Direction of effect (narrative)	Certainty of evidence (GRADE)	Main reasons for downgrading
Depigmentation improvement (short-term, ≤1-6 months)	2 (38)	Both techniques appeared clinically effective, with no clear consistent advantage for either arm at early follow-up where reported [16]	Low	Serious RoB (overall high/moderate study limitations); serious imprecision (small n; limited quantitative detail)
Repigmentation/recurrence (medium to long term)	2 (38)	Recurrence signals varied by follow-up: no recurrence at 1-6 months in one trial, but substantial repigmentation at longer follow-up in another [14,16]	Very low	Serious RoB; serious inconsistency (follow-up windows and recurrence patterns differ); serious imprecision

Discussion

Principal Findings

This systematic review indicates that electrosurgery/electrocautery is a clinically effective option for gingival depigmentation, as most comparative studies reported a clear short-term reduction in visible pigmentation after treatment. However, the findings also show that success cannot be judged only by immediate color change. In esthetic periodontal procedures, the overall clinical value of a technique depends equally on postoperative pain, quality of healing, and the stability of depigmentation over time. On that basis, electrosurgery appeared useful and practical, but its performance was not uniformly superior across all outcomes or comparators.

One of the main reasons for cautious interpretation is the heterogeneity of the available evidence. The included trials used different pigmentation indices, different extents of treated gingiva, and different follow-up periods, which made direct comparison difficult. Even so, a consistent pattern was seen: electrosurgery produced satisfactory early depigmentation because it removes the pigmented epithelial layer while also providing coagulation and good hemostasis [5,7]. This intraoperative control is a practical advantage over scalpel scraping, especially in the anterior esthetic zone where bleeding can reduce visibility. For that reason, electrosurgery remains a realistic clinical option in settings where laser units are not readily available.

Comparison With Other Depigmentation Modalities

At the same time, patient-centered outcomes tended to favor laser in several comparative trials. Recent evidence has similarly suggested that laser-based approaches may offer advantages in early postoperative comfort and bleeding control, although long-term superiority remains uncertain because outcome measures and follow-up protocols remain variable [21,22]. Studies by Chandna and Kedige and Yadav et al. reported lower early postoperative pain in diode laser groups, particularly during the first 24 hours, while Jagannathan et al. also noted more favorable early healing with laser-based treatment [13,14,20]. This difference is biologically plausible. Electrosurgery creates thermal injury in addition to epithelial removal, and when tissue contact is prolonged or repeated, lateral heat spread may increase inflammation, discomfort, and transient delay in epithelialization [23,24]. Therefore, the results of electrosurgery seem to be especially technique-sensitive, and operator control is likely to influence both healing and comfort.

Comparison with scalpel-based depigmentation showed a more balanced picture. Scalpel scraping remains simple, economical, and familiar to most clinicians, but it is associated with more intraoperative bleeding and may provide a less controlled field [25]. Electrosurgery offers better hemostasis and may reduce chairside difficulty, yet this benefit may come at the cost of greater early discomfort or slower superficial healing in some cases [7,15]. This suggests that neither modality is universally superior; rather, the choice depends on what is being prioritized clinically, such as affordability, hemostasis, patient comfort, or operator familiarity.

Repigmentation and Biological Interpretation

Repigmentation remains the most important long-term concern. The review found substantial variability in recurrence findings, largely because studies differed in follow-up duration and in how recurrence was reported. In practical terms, the short-term window in this review corresponded broadly to 1-6 months, whereas longer-term recurrence data were available only in a small number of studies extending to 12-15 months [14,15]. This is an important limitation because repigmentation is a time-dependent outcome and short follow-up may underestimate clinically meaningful recurrence. Jagannathan et al. demonstrated that repigmentation remains clinically relevant on longer follow-up, whereas shorter-term studies, such as Yadav et al., naturally reported fewer recurrent cases [14,20]. Therefore, early postoperative success should not be interpreted as evidence of durable long-term stability. Biologically, repigmentation may occur because of melanocyte migration from adjacent tissues or incomplete elimination of the basal pigmented layer [8,26]. However, this mechanistic explanation should be interpreted cautiously in the context of the present review, because most included studies did not directly evaluate biological pathways, only one study assessed melanocyte density histologically [19], and none directly measured melanocyte migration. Therefore, the biological explanation remains inferential rather than directly demonstrated by the included evidence.

Limitations and Implications for Future Research

The strength of these conclusions is limited not only by the methodological quality of the underlying studies but also by restricted external validity. Only eight studies were included, of which seven were randomized trials and one was a non-randomized study; most were small, single-center studies conducted in India in young adults with mainly physiological pigmentation of the anterior gingiva. Accordingly, the findings may not be directly generalizable to broader clinical populations with different age groups, ethnic backgrounds, pigmentation patterns, or treated sites. Several trials also had concerns related to randomization, concealment, blinding, and subjective outcome assessment, and the certainty of the evidence was downgraded mainly due to risk of bias and imprecision. In addition, the degree of heterogeneity across the included studies did not permit quantitative synthesis, so the conclusions of the review are based on structured narrative comparison rather than pooled effect estimation.

Overall, the present evidence supports electrosurgery as an effective and accessible depigmentation technique with clear practical advantages, but its postoperative experience and long-term stability appear more variable than immediate pigment reduction alone would suggest. Many included studies also incompletely reported operative parameters, including electrosurgical settings, waveform or power characteristics, electrode type, application intervals, laser wavelength and power parameters, and other procedural details. This limited reproducibility and made it difficult to determine whether variability in pain, healing, or recurrence reflected true differences between modalities or differences in technique execution. Future trials should use standardized measures of pigmentation and healing outcomes, clearly report procedural and device-related parameters, and include longer follow-up to enable more reliable comparisons across modalities.

## Conclusions

Electrosurgery is an effective method for achieving short-term gingival depigmentation and offers practical intraoperative advantages, particularly good hemostasis and clinical accessibility. Compared with other modalities, especially diode laser, it may be associated with less favorable early postoperative comfort and healing in some studies, while long-term stability remains uncertain because repigmentation appears to be time-dependent and is insufficiently evaluated in studies with extended follow-up. Based on the current evidence, electrosurgery can be considered a valid clinical option, but technique selection should remain individualized, and patients should be counselled that recurrence is still possible.

## Appendices

**Table 6:** Full search strings across all the included databases

Database	Date searched	Full electronic search string	Filters notes
PubMed	28 February 2026	((("Gingiva"[Mesh] OR gingiv*[tiab]) AND (pigment*[tiab] OR hyperpigment*[tiab] OR depigment*[tiab] OR melanosis[tiab] OR melanin[tiab] OR "oral pigmentation"[tiab] OR "gingival pigmentation"[tiab] OR "gingival depigmentation"[tiab] OR "gingival melanosis"[tiab])) AND ("Electrosurgery"[Mesh] OR electrosurg*[tiab] OR electrocauter*[tiab] OR diatherm*[tiab] OR electrofulgurat*[tiab] OR scalpel[tiab] OR laser*[tiab] OR "diode laser"[tiab] OR cryosurg*[tiab] OR "liquid nitrogen"[tiab] OR "free gingival graft*"[tiab] OR abrasion[tiab] OR scraping[tiab] OR stripping[tiab])) AND (randomized controlled trial[pt] OR controlled clinical trial[pt] OR comparative study[pt] OR random*[tiab] OR trial[tiab] OR comparative[tiab] OR controlled[tiab] OR cohort[tiab] OR "case-control"[tiab] OR "split-mouth"[tiab] OR "split mouth"[tiab]))	Searched from database inception to 28 February 2026; no date restriction applied at the search stage
Scopus	28 February 2026	TITLE-ABS-KEY(((gingiv* W/2 (pigment* OR hyperpigment* OR depigment* OR melanosis OR melanin)) OR "gingival pigmentation" OR "gingival depigmentation" OR "oral pigmentation" OR "gingival melanosis") AND (electrosurg* OR electrocauter* OR diatherm* OR electrofulgurat* OR scalpel OR laser* OR "diode laser" OR cryosurg* OR "liquid nitrogen" OR "free gingival graft*" OR abrasion OR scraping OR stripping) AND (random* OR trial OR comparative OR controlled OR cohort OR "case-control" OR "split mouth" OR "split-mouth"))	Document search; title, abstract, and keywords searched; inception to 28 February 2026
Embase	28 February 2026	(('gingiva'/exp OR gingiv*:ti,ab,kw) AND (pigment*:ti,ab,kw OR hyperpigment*:ti,ab,kw OR depigment*:ti,ab,kw OR melanosis:ti,ab,kw OR melanin:ti,ab,kw OR 'gingival pigmentation':ti,ab,kw OR 'gingival depigmentation':ti,ab,kw OR 'oral pigmentation':ti,ab,kw OR 'gingival melanosis':ti,ab,kw) AND ('electrosurgery'/exp OR electrosurg*:ti,ab,kw OR electrocauter*:ti,ab,kw OR diatherm*:ti,ab,kw OR electrofulgurat*:ti,ab,kw OR scalpel:ti,ab,kw OR laser*:ti,ab,kw OR 'diode laser':ti,ab,kw OR cryosurg*:ti,ab,kw OR 'liquid nitrogen':ti,ab,kw OR 'free gingival graft*':ti,ab,kw OR abrasion:ti,ab,kw OR scraping:ti,ab,kw OR stripping:ti,ab,kw) AND ('randomized controlled trial'/exp OR random*:ti,ab,kw OR trial:ti,ab,kw OR comparative:ti,ab,kw OR controlled:ti,ab,kw OR cohort:ti,ab,kw OR 'case control study'/exp OR 'case-control':ti,ab,kw OR 'split mouth':ti,ab,kw OR 'split-mouth':ti,ab,kw))	Emtree exploded where applicable; searched in title, abstract, and keywords; inception to 28 February 2026
Web of Science Core Collection	28 February 2026	TS=(((gingiv* NEAR/2 (pigment* OR hyperpigment* OR depigment* OR melanosis OR melanin)) OR "gingival pigmentation" OR "gingival depigmentation" OR "oral pigmentation" OR "gingival melanosis") AND (electrosurg* OR electrocauter* OR diatherm* OR electrofulgurat* OR scalpel OR laser* OR "diode laser" OR cryosurg* OR "liquid nitrogen" OR "free gingival graft*" OR abrasion OR scraping OR stripping) AND (random* OR trial OR comparative OR controlled OR cohort OR "case-control" OR "split mouth" OR "split-mouth"))	Topic search; searched from inception to 28 February 2026
ScienceDirect	28 February 2026	("gingival pigmentation" OR "gingival depigmentation" OR "oral pigmentation" OR "gingival melanosis" OR (gingiv* AND (pigment* OR hyperpigment* OR depigment* OR melanosis OR melanin))) AND (electrosurg* OR electrocauter* OR diatherm* OR electrofulgurat* OR scalpel OR laser* OR "diode laser" OR cryosurg* OR "liquid nitrogen" OR "free gingival graft*" OR abrasion OR scraping OR stripping) AND (random* OR trial OR comparative OR controlled OR cohort OR "case-control" OR "split mouth" OR "split-mouth")	Boolean search using platform-supported operators and parentheses; searched from inception to 28 February 2026
EBSCOhost	28 February 2026	TX (("gingival pigmentation" OR "gingival depigmentation" OR "oral pigmentation" OR "gingival melanosis" OR (gingiv* N2 (pigment* OR hyperpigment* OR depigment* OR melanosis OR melanin))) AND (electrosurg* OR electrocauter* OR diatherm* OR electrofulgurat* OR scalpel OR laser* OR "diode laser" OR cryosurg* OR "liquid nitrogen" OR "free gingival graft*" OR abrasion OR scraping OR stripping) AND (random* OR trial OR comparative OR controlled OR cohort OR "case-control" OR "split mouth" OR "split-mouth"))	Advanced search in EBSCOhost interface; relevance sorting used; first 300 results screened, where applicable
Google Scholar	28 February 2026	"gingival pigmentation" OR "gingival depigmentation" OR "oral pigmentation" OR "gingival melanosis" electrosurgery OR electrocautery OR diathermy scalpel OR laser OR cryosurgery OR "liquid nitrogen" OR "free gingival graft"	Results sorted by relevance; first 300 results screened manually
Semantic Scholar	28 February 2026	("gingival pigmentation" OR "gingival depigmentation" OR "oral pigmentation" OR "gingival melanosis") AND (electrosurgery OR electrocautery OR diathermy) AND (scalpel OR laser OR cryosurgery OR "liquid nitrogen" OR "free gingival graft" OR abrasion OR scraping OR stripping)	Results sorted by relevance; first 300 results screened manually
